# Validation of Small-Animal Look-Locker Inversion Recovery (SALLI) Cine versus Cine MR in assessment of left ventricular function

**DOI:** 10.1186/1532-429X-15-S1-P9

**Published:** 2013-01-30

**Authors:** Darach O h-Ici, Sarah Jeuthe, Hubertus Pietsch, Gunnar Schuetz, Felix Berger, Thore Dietrich, Sebastian Kozerke, Titus Kuehne, Daniel Messroghli

**Affiliations:** 1 Congenital Heart Disease and Pediatric Cardiology, Deutsches Herzzentrum Berlin, Berlin, Germany; 2 Internal Medicine-Cardiology, Deutsches Herzzentrum Berlin, Berlin, Germany; 3 Institute for Biomedical Engineering, University and ETH Zürich, Zürich, Switzerland; 4 Imaging Sciences and Biomedical Engineering, King's College London, London, UK; 5 MR and CT Contrast Media Research, Bayer Pharma AG, Berlin, Germany

## Background

Small-Animal Look-Locker Inversion Recovery (SALLI) is a novel imaging method that generates simultaneously cardiac T1 maps, cine MR and inversion recovery-prepared images for high heart rates. T1 mapping by SALLI has been tested and compared with histology, however the assessment of left ventricular (LV) function has not yet been validated.

Our aim was to compare the measurement of LV function between the SALLI sequence and the standard Cine MRI sequence.

## Methods

8 healthy rats and 4 rats with surgically induced myocardial infarction were imaged. MRI was performed on a whole-body 3.0T MR unit with a 70 mm solenoid coil for rats. After generation of survey images and of a long-axis set of cine images, a stack of LV short-axis cine images was acquired (phases 30, TR 6.8 ms, TE 3.3 ms, flip angle 15°, field of view 80 x 64 mm, acquired voxel size 0.4 x 0.4 x 1.5 mm, number of signal averages 3, slices 7, inter-slice gap adjusted to allow for coverage of the entire LV; range -0.5 to -0.4 mm).

Short axis SALLI MR imaging was then performed in the same 7 short axis orientations from apex to base. Typical SALLI parameters were as follows: 64 x 64-mm field of view, 0.60 x 0.60 mm pixel size, 3.0 mm- thick sections, 5.2/2.2, 10° flip angle, 4000-msec AD, 4000-msec RD, 12 phases (i.e., temporal resolution of 16.7 ms at 300 beats per minute), temporal undersampling factor of two, three signals acquired, and acquisition time of 8 minutes 30 seconds per slice.

Images were transferred to a cardiac MRI analysis software package and analysed in a blinded fashion to assess the ejection fraction from the stack of short-axis cine slices and the short axis SALLI slices. For each of the sets of data the image quality was visually assessed (good, no artifacts=1, minor artifacts=2, non-evaluable, major artifacts=3).

**Figure 1:**
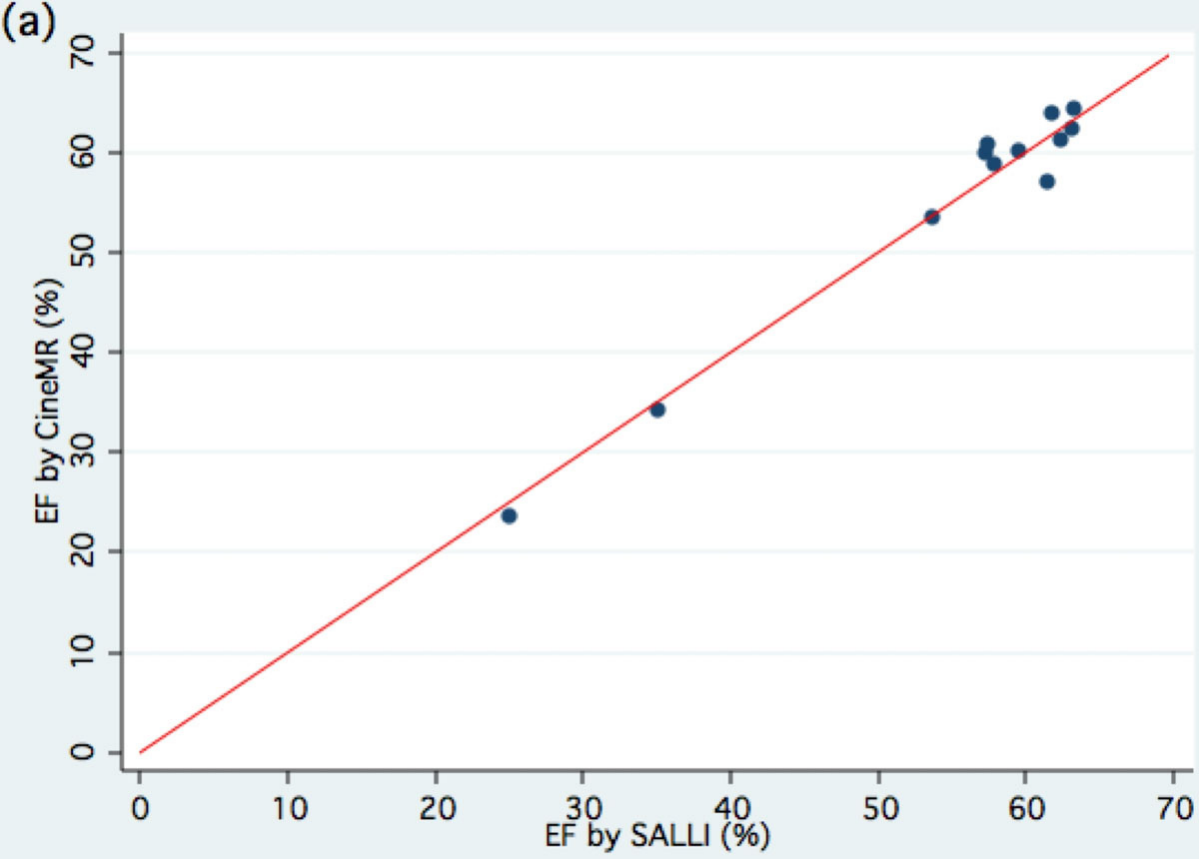
Scatter plots with line of agreement between EF% by Cine-MR and SALLI

**Figure 2:**
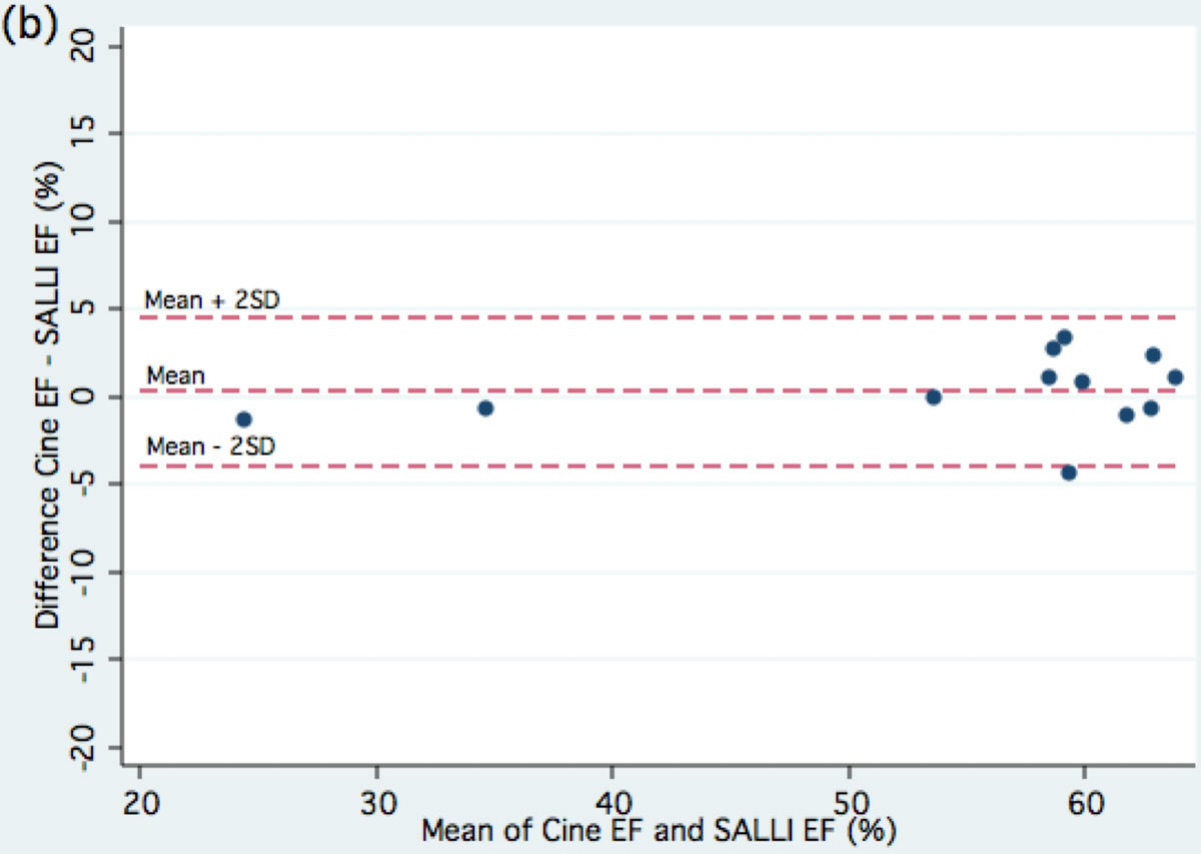
Bland-Altman plot for comparisons between techniques

## Results

Data was available for 12 Rats. Image quality was good or adequate in all cases and was not significantly different (p=0.56) between cine-MR (1.2 ± 0.4) and SALLI (1.3 ± 0.5). Left ventricular function as measured by both methods was very similar.

EF by cine MR was 55.1 ± 3.7% and by SALLI it was 54.8 ± 3.5%. No significant difference was found between the methods of measurement (mean difference 0.3 ± 2.1%, p=0.63).

## Conclusions

The aims of this study were to validate the accuracy of SALLI cine imaging versus standard MRI cine imaging in the assessment of left ventricular function. Both methods provided similar results across a range of ventricular function and suggest that SALLI cine provides comparable measurement of left ventricular function. Image quality was similar between both methods and interpretable in all cases.

As SALLI generates multimodal (Cine MR, T1 maps and Inversion-Recovery prepared) images from the same dataset, this may not only save time, but also allow comparison of findings without the need for further image registration.

## Funding

Dr O h-Ici is funded through a "Sachmittelbeihilfe" granted to Dr Messroghli by the Deutsche Forschungsgemeinschaft. Drs Kuehne and Messroghli are supported through the German Federal Ministry of Education and Research (grants 01EV0704 and FKZ01G10210, 01GI0601).

